# Microscopic Analysis of Current and Mechanical Properties of Nafion^®^ Studied by Atomic Force Microscopy

**DOI:** 10.3390/membranes2040783

**Published:** 2012-11-16

**Authors:** Renate Hiesgen, Stefan Helmly, Ines Galm, Tobias Morawietz, Michael Handl, K. Andreas Friedrich

**Affiliations:** 1 University of Applied Sciences Esslingen, Kanalstrasse 33, Esslingen 73728, Germany; Email: ines.galm@hs-esslingen.de (I.G.); tobias.morawietz@hs-esslingen.de (T.M.); michael.handl@stud.hs-esslingen.de (M.H.); 2 German Aerospace Center, Institute of Technical Thermodynamics, Pfaffenwaldring 38-40, Stuttgart 70569, Germany; Email: stefan.helmly@dlr.de; 3 Institute for Thermodynamics and Thermal Engineering, University of Stuttgart, Stuttgart 70550, Germany

**Keywords:** Nafion, AFM, current, adhesion, stiffness, nanostructure, model

## Abstract

The conductivity of fuel cell membranes as well as their mechanical properties at the nanometer scale were characterized using advanced tapping mode atomic force microscopy (AFM) techniques. AFM produces high-resolution images under continuous current flow of the conductive structure at the membrane surface and provides some insight into the bulk conducting network in Nafion membranes. The correlation of conductivity with other mechanical properties, such as adhesion force, deformation and stiffness, were simultaneously measured with the current and provided an indication of subsurface phase separations and phase distribution at the surface of the membrane. The distribution of conductive pores at the surface was identified by the formation of water droplets. A comparison of nanostructure models with high-resolution current images is discussed in detail.

## 1. Introduction

Proton-conducting membranes are one of the key components in polymer-electrolyte fuel cells. The most important type of membrane is Nafion^®^. This basic function is the result of the Nafion^®^ molecular structure—A perfluorinated ionomer with negatively charged sulfonic groups on its side chains. The Teflon-like backbone of the molecule provides excellent stability with respect to the chemical environment and the electrochemical conditions in a fuel cell. The molecule has a comb-like structure and, in a self-assembling process during solidification, phase-separation morphology of discrete hydrophobic and hydrophilic regions occurs. The hydrophilic phase provides an ionically conductive water-filled network for proton conduction. This microstructure strongly influences the hydration and thereby the conductivity of the membrane. 

Although the detailed phase-separated structure of Nafion^®^ is still a matter for continuous study, it is well known that the microstructure of Nafion^®^ strongly depends upon the hydration state, the temperature, and the current flow. These morphological changes can be fast, such as the restructuring of the surface in contact with liquid water within seconds, or slow, such that achieving a steady state in the bulk can require days or even months [1]. 

Most of the Nafion^®^ structural models are primarily based on the Eisenberg description, which considers ionic clusters dispersed in a hydrophobic polymeric matrix to reduce the total free energy of the system [2]. These models have been used to describe the strong water swelling behavior of Nafion and the pronounced dependence of conductivity on water content. However, more recently, the importance of the polymer interface has been recognized for its properties. Several studies have suggested that the rate-limiting step for water transport is interfacial transport at the interface [3,4]. Inside the conductive network, a finite pressure is present, and the state of hydration is a balance between different forces. One force is the osmotic pressure due to sulfonic groups, and a second force is the elastic pressure due to visco-elastic deformation of the matrix. The micro-phase separation is stabilized by the inner surface tension, which leads to an additional Laplace pressure [5,6].

Microstructure has been the subject of intensive investigations. One of the first detailed studies that focused on the morphological structure of the PEM was performed by Gierke *et al.* [7,8,9]. Gierke’s cluster model suggested the existence of connected globular ionic clusters and could explain the percolation threshold at a water volume fraction greater than 20%. Recently, parallel cylindrical water nano-channels (2008) were deduced from small-angle X-ray scattering (SAXS) experiments, which could explain the structural reorganization as water content increases, and were in agreement with the observed transport properties. The existence of crystallites was also taken into account [10]. From the results of small-angle neutron scattering (SANS) experiments, another structural model for Nafion^®^ was proposed by Gebel *et al.* that describes the membrane as an aggregation of polymeric chains that form elongated objects (simplified as cylinders) embedded in a continuous ionic medium. On a larger scale, these aggregates form bundles, which are characterized by an orientation order between the aggregates [11,12]. In this model, the water medium is described as a 3D continuous medium (as a function of water sorption) rather than confined water molecules in a channel (1D) and an inverted micelles network [13]. From SAXS analysis at wide-, small- and ultra-small angles, a ribbon-like structure of polymer particles was concluded; these particles are assembled in bundles of approximately 50 nm [14,15]. The models of Schmidt–Rohr and Gebel are contradictory in a simple aspect: the first author predicts polymer channels that contain water inside the structure, whereas the second author predicts polymer rods surrounded by water. In this latter model, the polymer structure typically changes from a water-containing polymer network at low water content to a structure-inversed polymer in the water phase [11]. Numerous investigations on these membranes have been published and have been summarized in review articles [16,17,18].

The nanostructure of Nafion^®^ has been investigated intensively as a function of the temperature and the water content; however, the investigations of structural aspects under current flow are rare. From the complex behavior of Nafion^®^ known from equilibrium states without current flow, strong influences on the structure can be expected for the proton conduction associated with water flow. The strength of conductive atomic force microscopy (AFM) in this regard is that it provides direct information under current flow, which is highly relevant for fuel cell applications. However, this direct information is derived from the interface properties, and this aspect is therefore discussed below. 

**Figure 1:**
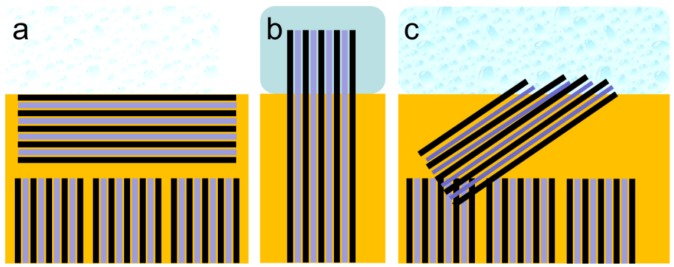
(**a**) Drawing of the equilibrium structure of the Nafion^®^–water vapor interface with bundles of Nafion^®^ fibrils parallel to the interface forming an interface layer several nanometers thick without connection to water vapor at the surface according to Bass *et al.* [6]. (**b**) Drawing of the equilibrium structure of the Nafion^®^–water interface with bundles of Nafion^®^ fibrils vertical to the interface with connection to the water phase according to Bass *et al.* [6]. (**c**) Scheme of the non-equilibrium structure of Nafion^®^ measured under current at the water/vapor interface.

The structure of the interface is critically important for the conductivity of a fuel cell because it connects the membrane to the electrodes. The interface structure depends on the surface energy of the membrane and the contact material. The interfacial area of Nafion^®^, the fraction of hydrophilic surface, and thereby the total interfacial energy are closely related to its nanostructure [5]. The interface structure of Nafion^®^ in contact with liquid water and water vapor has been investigated in detail by Bass *et al.* with respect to the orientation of the assumed hydrophilic water-filled polymer fibrils [5,6]. The polymer strands are assumed to be present as bundles aligned parallel to a solid/gas interface or tend to be perpendicular at the liquid-water interface. The parallel-aligned hydrophilic channels in a lamellar structure do not allow easy water penetration into the membrane. In contrast, at the membrane/liquid-water interface, these bundles are reported to reorientate in the direction perpendicular to the membrane surface, at which point water can enter the widened and open hydrophilic conductive channels. Based on contact-angle measurements taken at different interfaces, the fraction of hydrophilic surface area has been calculated to change from 0.05 in the dry polymer to greater than 0.11 in water vapor and up to 0.95 in liquid water [5,6]. A scheme of this interface interaction is given in Figure 1.

A change in connectivity in the conductive network depending on water activity was observed based on tortuosity measurements. At low water activity (λ < 4), the connectivity of paths across the membrane was low, which indicated a high tortuosity. At high water activity, the hydrophilic domains were swollen from water sorption and connected more parts of the network [3]. 

In a working fuel cell, the situation is further complicated because the membrane state is strongly affected by the current flow and the operating conditions. With current flow, water is transported by electro-osmotic drag through the membrane from the anode to the cathode, which significantly changes the local water content inside the membranes and at the surface. At the surface of the cathode side, additional reaction water is produced by the electrochemical reaction. Instead of equilibrium, a steady state is established, which changes with time during dynamic operation. The interface where the reaction is assumed involves not only water but also a platinum surface, which might lead to a different surface structure of Nafion^®^ bundles compared to a pure water interface. 

In this paper, we report two novel aspects for Nafion^®^ studies that are related to the conductive AFM and the application of material-sensitive AFM analysis by advanced methodology, specifically, the so-called PeakForce-QNM^©^ and PeakForce-TUNA^©^ (Bruker Corp.). With a material-sensitive AFM and a catalytically active platinum-coated AFM tip, the analysis of local conductivity and the interface structure is analyzed under current flow. We therefore obtain additional local information related to mechanical heterogeneity and, in addition, information related to how this heterogeneity evolves and is affected by current flow. Even if there is still no perfect match to the conditions in a working fuel cell, this investigation is more relevant for the situation in a fuel cell compared to the common *ex situ* methods. During current measurement, a contact of the platinum-coated tip provides the same platinum-membrane interface that is present in a fuel cell catalyst particle. Because platinum has a hydrophilic surface [19,20,21], the interface properties are comparable, and liquid water is most likely present. In the AFM measurements, the contact time between the Nafion^®^ surface and the platinum tip is short and may not provide sufficient time to evolve the equilibrium structure of a membrane-platinum interface according to the interfacial energy of this configuration. We report the simultaneous measurements of the topography, conductivity, and mechanical properties of Nafion^®^ 112 membranes for different situations. For example, membranes were analyzed by AFM after being stored in water without an initial current flow and after an activation of the membrane in an electrolysis arrangement.

## 2. Experimental Section

### 2.1. Sample Preparation

**Figure 2:**
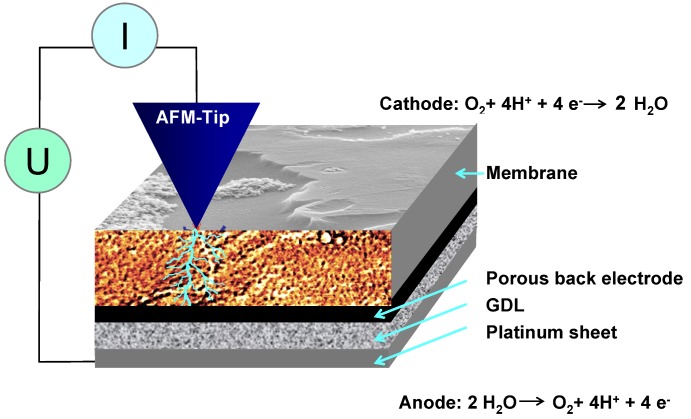
Schematic drawing of a conductive atomic force microscopy (AFM) measurement at an ionically conductive membrane in an ambient humid environment: Ionic current through the membrane results from two electrochemical reactions at the extended back electrode and the platinum-coated AFM tip.

As solid-electrolyte membranes, protonated Nafion^®^, mostly of type 112, was used. For current measurements, the back side of fully protonated membranes was coated with an extended porous electrode that contained platinum. Some samples were single-side coated with a porous PT/C/Nafion^®^ electrode via dry hot spraying at DLR. The membranes were stored for at least one week in ultra-pure H_2_O with H_2_SO_4_ added to prevent bio-deterioration. The measurements were performed in a humid environment in ambient air using an applied voltage greater than 1.3 V. The current flow is based on two electrochemical reactions: Mainly oxygen evolution from water splitting at the back electrode and oxygen reduction with the formation of water at the platinum-coated AFM tip, which is used as a nano-electrode at the otherwise bare membrane surface. It cannot be excluded that hydrogen evolution may take place in a low rate at the tip but this is not expected to alter the current mapping as confirmed by changing bias voltage. A scheme of the setup is given in Figure 2. For AFM analysis, the electrode-coated samples were bonded to Toray paper using a liquid Nafion^®^/graphite mixture that contained 0.5 mg/cm^2^ of platinum. Unless otherwise stated, the samples were activated in an electrolysis arrangement between a platinum sheet and a platinum grid as electrodes for at least 1.5 h at a voltage of 2.7 V in water. For AFM measurements, the complete sample was placed on a platinum sheet, and the surface was dried with a tissue to remove excess water. The bias was applied to the cell such that hydrogen evolution takes place at the side with the carbon components; thereby carbon corrosion is avoided.

### 2.2. AFM Measurements

**Figure 3:**
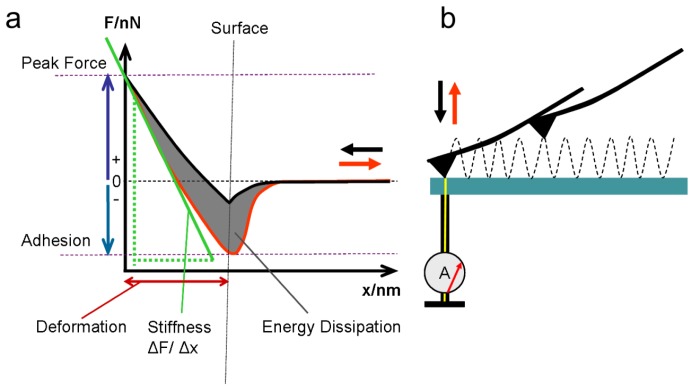
(**a**) Evaluation of mechanical properties from the force-separation curve; (**b**) The force-separation curve and evaluation of mechanical properties at every image point. Current measurement with a conductive AFM tip is also possible during contact.

The measurements were performed on a Multimode 8 AFM (Bruker Corp.) that provided an advanced tapping-mode utility with additional current measurement, the “PeakForce-TUNA^TM ^mode”, where mechanical properties can be evaluated together with topography data and the local ionic current. The tip vibrates vertically at a fixed frequency of 2 kHz towards the sample and, within each cycle, touches the surface with an ultralow force. During approach and retraction, force-distance curves are recorded at each image point, and, together with the topography, the following mechanical properties are evaluated (Figure 3): adhesion force (smallest force during detachment of the tip from the surface), energy dissipation (work), peak force (at the position of tip reversal), stiffness (linear fit to the tip reversal curve in Derjaguin–Müller–Toporov mode, abbreviated as DMT modulus [22]), deformation (impression depth), and phase shift (the phase between the applied oscillation and the cantilever oscillation) (Figure 3). The instrument and its analysis software construct a map of each property as an image. With the conductive platinum-coated tip, the current during contact can be measured at each point in addition to the mechanical properties. Previously, conductivity was typically measured in contact mode [23,24,25,26]. Measurements in contact mode apply a high pressure to the surface and lead to significant deformation of the sample, as shown previously by Buratto [27]. In TUNA mode, the current measured at each contact point is analyzed by a built-in lock-in amplifier, and the averaged steady-state current (referred to as the TUNA current) is measured. Without the lock-in amplifier, the current during quantitative nano-mechanical (QNM) tapping can also be measured.

### 2.3. Calibration of Adhesion Force and Energy Dissipation

Statistical determination of the surface properties was performed through the evaluation of measured image data. From the maps, a histogram can be calculated that displays the frequency of occurrence of, e.g., the adhesion force value from the data of a complete image or a certain area of an image. The peak position in the histogram was taken as the average for the analyzed area. The measurements were performed under ambient conditions at room temperature and at a relative humidity (RH) of 40%–50%. A mean value, including error bars, was calculated from the current images measured under same conditions. All images used for the evaluation of sample properties were recorded under identical conditions with the same image size. 

## 3. Results and Discussion

### 3.1. Activation of Protonated Nafion^®^

**Figure 4:**
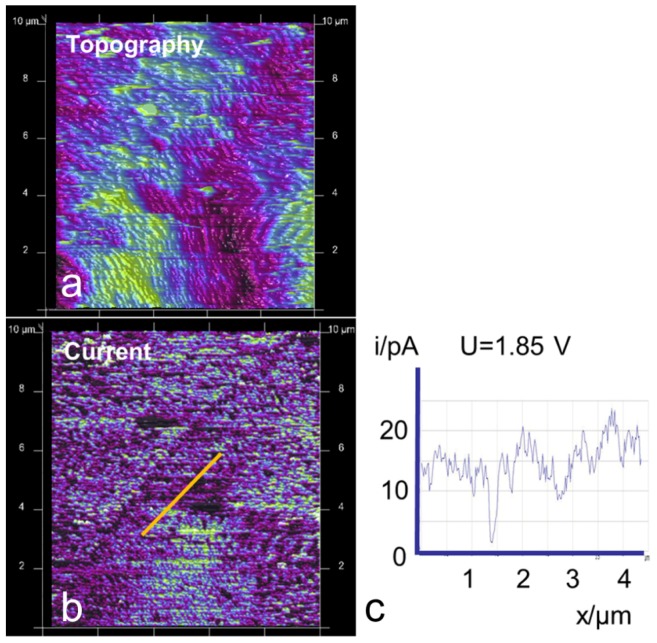
(**a**) AFM image of Nafion^®^ 112 before activation with a lamellar surface structure; differently orientated domains are visible; image size 10 µm × 10 µm. (**b**) Map of ionic current at the same area at U=1.85 V. (**c**) The current profile measured along the marked line in the current image shows small current values, of the order of 10–20 pA.

At a protonated and water-soaked but not activated Nafion^®^ sample, the conductivity measured by conductive AFM is barely detectable. The common activation procedure is to force a flow of current by, e.g., electrolysis. Without activation, even after more than one week of storage in water, only a current of a few pA could be detected in all cases (Figure 4). The often-observed lamellar structure in the topography, which is also visible in the conductivity images (Figure 4), supports a parallel orientation of polymer structures with respect to the surface, as has been reported by other researchers [5,6,28]. A voltage of U = 1.85 V was used because of the relatively low current response of the membrane.

**Figure 5:**
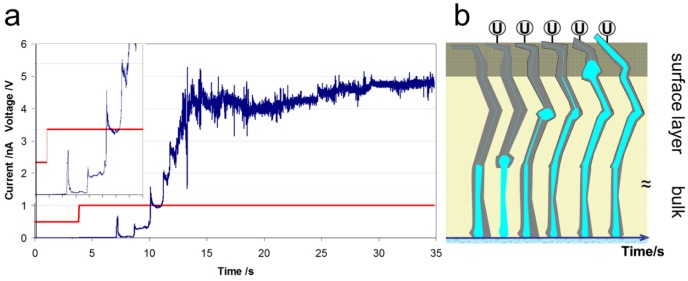
(**a**) Dependence of current with time after a voltage step from 0.5 to 1.0 V. In the inset, the onset region for the step-wise increase in the current is visible. The level of the pseudo-steady-state current is increased after each subsequent step. (**b**) Scheme of the stepwise widening of current/water channels under water pressure due to electro-osmotic drag of water by protonic current according to the visco-elastic properties of Nafion^®^.

We have previously observed that the conductivity of a Nafion membrane can be activated significantly by forcing a current through the membrane electrode assembly [24]. From local impedance measurements with the AFM, a successive humidification of a Nafion^®^ membrane after subsequent measurement of I(U) curves at the same position was concluded [24]. To understand this process, in a separate experiment a voltage step from 0.5 to 1.0 V was applied to a non-activated membrane in the AFM configuration, and the conductivity image was recorded for 35 s. During this time, the stepwise evolution of higher currents to a steady-state value was detected, as shown in Figure 5a. A stepwise increase in the current from the low pA range to the 4 nA range is visible with steps in a time interval of 1–2 s. A current step is characterized by an overshoot of current followed by a smaller decrease to a higher current level than before. The time interval between two subsequent steps decreases with time. Nafion^®^ is a visco-elastic polymer that reacts to mechanical pressure with only a partially elastic size elongation. During storage in water, water diffuses into the conductive network of the membrane, which swells, and the detection of a subsequent ionic conductivity would be expected thereafter. The observation of the ultra-low current flow in fresh samples by AFM can be explained by the existence of a different surface structure that inhibits conductivity, as proposed by several authors, e.g., Bass *et al.* [5,6]. These authors derived this interpretation from surface energy considerations and grazing-incidence small-angle X-ray scattering (GISAXS) experiments and concluded, based on time-dependent SANS measurements, that preferentially aligned micelles were present. Gebel *et al.* concluded that the sorption kinetics were dominated by the transfer at the membrane interface [29]. 

We interpret the activation process and the AFM observations in the context depicted in Figure 5b. The application of a voltage between the AFM tip and the extended back electrode leads to a proton current, which is accompanied by water flow due to the electro-osmotic drag. The water flow is most likely hindered by a surface layer with limited conductivity, and the water flow driven by the current leads to an increase in water pressure at a bottleneck in the conductive network. However, a change in connectivity of the conductive network in the bulk of the membrane, which has been reported to be dependent on water activity, may also occur [3]. At a certain water pressure, the inner pressure of the polymer is overcome, and a widening of the water channel occurs, which leads to a sudden current increase. The subsequent release of static water pressure associated with water flow is followed by elastic release of the polymer and shrinkage of the channel. Because of the irreversible plastic deformation of the polymer, a permanent widening of this conductive channel occurs and results in a higher steady-state current. This cycle was repeated with decreasing time intervals, and smaller current changes (*i.e.*, smaller built-up pressures) were observed until a final current level was reached. This activation is assumed to lead to a preferentially orientated conductive network in the surface layer. The structure induced by current flow with an applied voltage results in a water-filled channel that is sufficiently large to fulfill the hydrodynamic water flow requirements. Bulk water properties are also associated with high proton conductivity according to the Grotthuss mechanism [30]. The energy input by the applied voltage and the forced current flow obviously accelerates the reconstruction, at least at the surface layer, compared to the change observed for equilibrium conditions at a liquid water interface. In a fuel cell, an activation time is also observed, and optimum performance is typically reached only after some hours of operation, which may be caused, in part, by the necessity of a similar membrane activation process. This process obviously occurs at start-up of a fuel cell after the application of voltage and current flow.

For the following AFM measurements, all samples were activated in an electrolysis arrangement before they were measured.

### 3.2. AFM Measurements of Topography and Current

**Figure 6:**
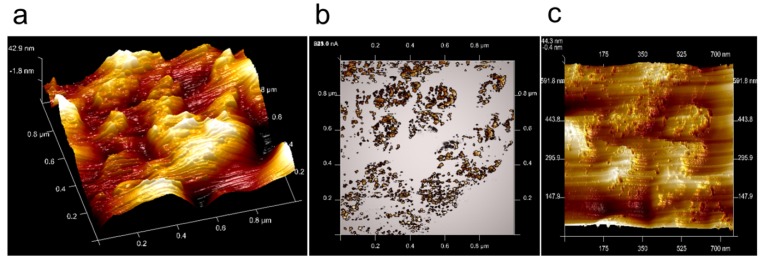
(**a**) AFM image of Nafion^®^ 112 under current flow with a 3-dimensional view of the topography after the Nafion was activated in an electrolysis setup; the image size is 1 µm × 1 µm. (**b**) Simultaneous measured current image; the current values are of the order of 300–600 nA. (**c**) Overlay of the current image onto the topography. Current flow is observed at edges of the swollen regions.

The surface topography of Nafion^® ^112, imaged by TUNA-mode tapping AFM where the Nafion is fully humidified and under current flow, is shown in Figure 6a. The maximum height difference was approximately 60 nm on the image length of one micrometer. Several protrusions are visible with a size of approximately 200 nm; the protrusions are composed of smaller features with a diameter of approximately 50 nm. 

**Figure 7:**
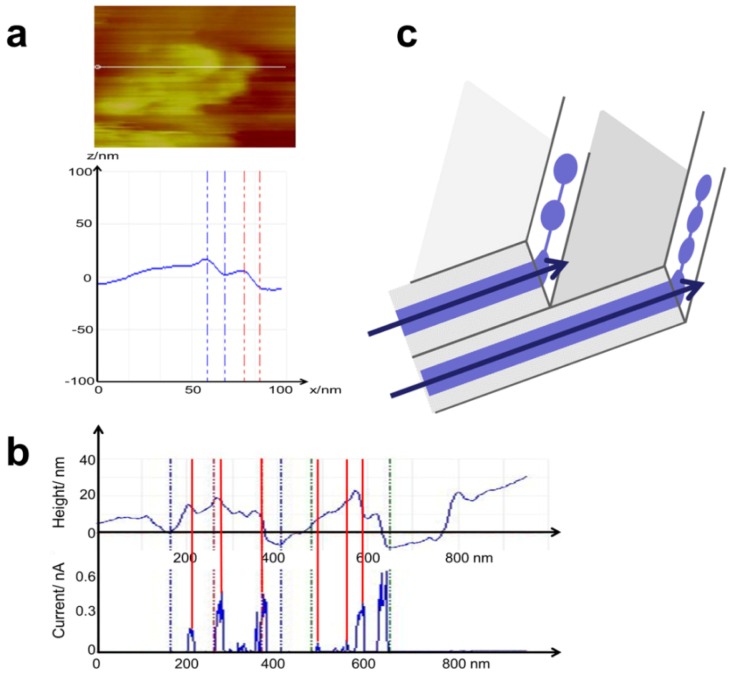
(**a**) Zoom of the AFM topography image in Figure 6 that shows one of the protrusions. Two steps are visible in the profile with a step height of approximately 14 nm (equidistant scale) and a tilt angle of 10° (a measurement of 15 similar features gives a mean angle of 13° ± 3°). (**b**) Comparison of the surface topography and the current profile measured in Figure 6. Current flow is restricted to the edges of the steps. (**c**) Schematic drawing of the steps with tilted water/current channels.

The simultaneously measured current image is given in Figure 6b. The current flow occurs predominantly at the larger protrusions and is increased at their edges. Current spots with a magnitude of approximately 1 nA are mostly aligned in the curvatures of the protrusions. For better identification of the conductive sites, the current image has been subtracted from the topographical image. Current flow is indicated by the black spots in Figure 6c. The protrusions exhibit a gentle increase on one side and decrease in steps. In the profile line (Figure 7a) through one of these protrusions, two steps with a width of approximately 10 nm and a height of approximately 14 nm are visible (equal scale in *x* and *z*). Most of the other steps have a height of approximately 10 nm. Current flow is located mainly at the edges of the protrusions and is detected directly at the vertical sides of the steps. Step height is approximately 14 nm, as visible in the direct comparison of height and current profile lines in Figure 7b. Based on model considerations, a size of 6–8 nm [30,31] is proposed for a structure of two hydrophobic layers and an interior water channel connected to sulfonic acid groups at the polymer side chains. The measured step heights at the surface are twice this value. This discrepancy may arise from the fact that these values were calculated for bulk Nafion^®^ in equilibrium. Under current flow, due to the hydrostatic water pressure, the height may deviate from the calculated size of a conductive channel.

### 3.3. Mechanical Properties

Under equilibrium conditions, the orientation of Nafion^®^ at the surface depends on the total surface energy of the system, including the neighboring material at the interface. At a dry interface, which can be assumed to be present under laboratory ambient conditions, the outermost surface exposes the hydrophobic polymer backbone with PTFE-like properties; consequently, the hydrophilic fraction accounts for only 11% of the exposed area [5]. A (water) wetted interface has a hydrophilic surface due to the presence of polymer side groups with sulfonic acid groups with a fraction of 95% hydrophilic area according to Bass *et al.* [5].

**Figure 8:**
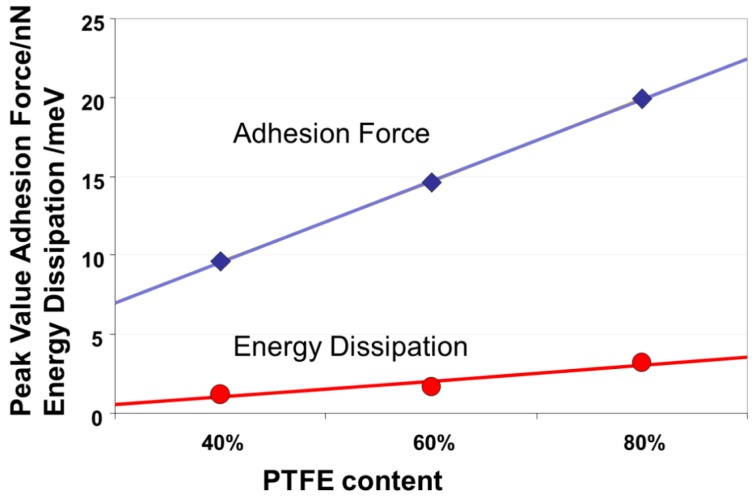
Dependence of adhesion force derived from samples with a known carbon/PTFE composition measured using PeakForce-QNM^®^ under dry conditions.

The dependence of adhesion forces and energy dissipation measured by AFM has been investigated and calibrated using a series of samples prepared with various controlled ratios of Teflon (PTFE) to carbon. These results are used as an analogy to better understand the increase of adhesion in the Nafion^®^ polymer. These samples were used as a model system with different amounts of hydrophobic PTFE (as found in the backbone of Nafion^®^ molecule) and carbon at the surface. They were analyzed by AFM in QNM^TM^ tapping mode in ambient air under ambient (dry) conditions. The statistical evaluation of the mean energy dissipation and the mean adhesion force as a function of the amount of PTFE with a confidence level 95% leads to a linear increase in both properties with increasing PTFE (Teflon) content, as shown in Figure 8 [23].

**Figure 9:**
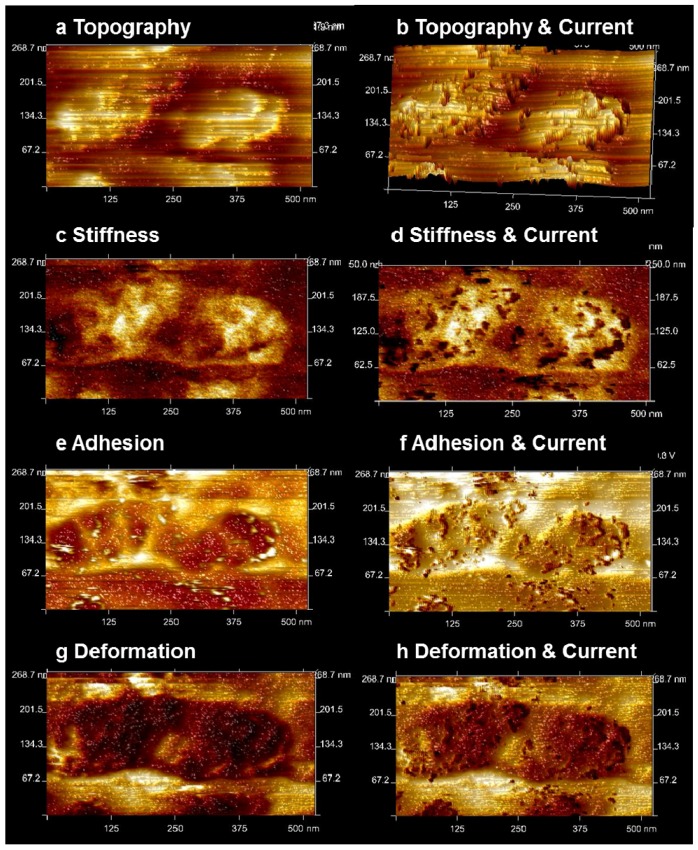
Zoomed area of Figure 6 of activated Nafion^®^ 112 under current flow. (**a**) Topography image of two swollen areas. (**b**) Overlay of topography and current images; current flow is restricted to step edges. (**c**) Conductive swollen areas have high stiffness. (**d**) Overlay of stiffness image with current; decrease of stiffness at site of current spots. (**e**) Decreased adhesion at swollen area with high adhesion at small spots. (**f**) Overlay of adhesion image with current; high adhesion spots coincide with larger current spots and indicate the formation of water droplets. (**g**) Deformation is decreased at the swollen area. (**h**) Overlay of the deformation with the current image; deformation is mostly reduced at the positions of water droplets.

In Figure 9, starting from the top, the topography (a), the DMT stiffness (b), the adhesion force (c), and the deformation (d) in a smaller area (500 × 500 nm) of Figure 6 are shown on the left side. 

**Figure 10:**
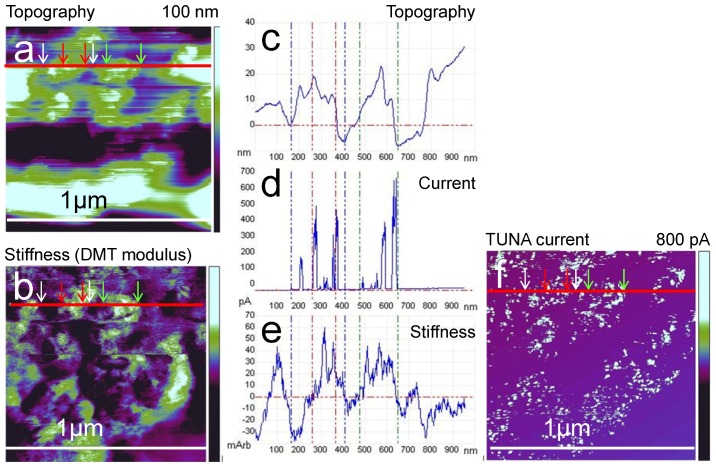
AFM measurement of the membrane in Figure 6 with mapping of topography (**a**), stiffness (**b**), and profiles of the topography (**c**) and current (**d**) of the membrane from Figure 7b with the addition of the stiffness profile (**e**) along the same line; the stiffness values are enhanced at swollen regions. The stiffness is slightly decreased directly at the site of current flow (compare with (**d**) and (**f**)).

On the right side, in Figure 9b,d,f,h, the current signal is subtracted from the left image, and the sites where current is detected are visible as black spots. In the topography image (Figure 9a), two of the larger protrusions (approximately 150 nm in size) are imaged; as previously discussed; the current flow is mainly located at the margins of the protrusions, and most of the current is recorded directly at the vertical steps. Notably, spots of very high adhesion (white spots Figure 9f) are observed, which are in the vicinity of the current signal. On the left side of Figure 10b, the stiffness signal of this area is given. The protrusions have a stiffness that is clearly higher than that of the surrounding area and thereby distinguishable. Inside the area of the protrusions, the stiffness signal also varies. On the right side, where the current has been subtracted from the stiffness image, the greatest stiffness is measured just between the current spots. Interestingly, the stiffness is somewhat lower directly at the position of the current spots. The adhesion force in Figure 9e, left side, exhibits a significantly smaller value at the protrusions. As previously mentioned, a few bright spots with very high adhesion are visible. A comparison of the conductive spots with the adhesion signal (right side) shows a coincidence of the white spots with the larger current spots. The whole area exhibits a small deformation (Figure 9g), with the smallest values at the position of the current spots visible at the right side. This mechanical property (high adhesion and low deformation) is explained by the small compressibility of liquids such as water, which obviously cannot be squeezed by the AFM tip during the short contact time. Therefore, we interpret the high adhesion spots as water droplets that result from the current flow and the associated water transport. For a direct comparison, the profile lines of topography, current, and stiffness (DMT) are given in Figure 10. 

**Figure 11:**
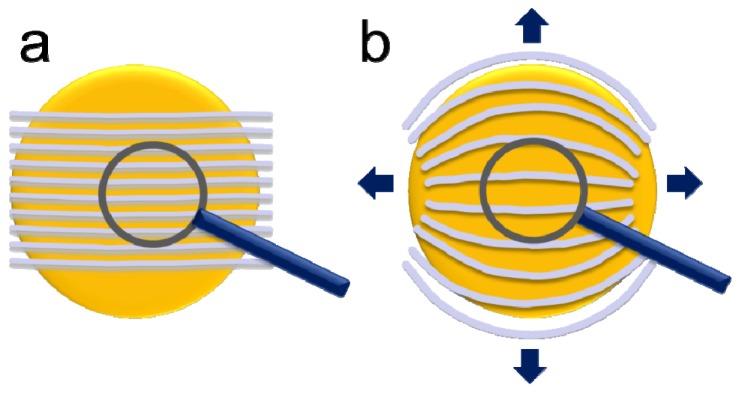
(**a**) Scheme of the density of polymer backbones of a flat surface. (**b**) After swelling of the surface area, the density of the polymer backbone is decreased.

Areas with distributed conductive spots and those without any current are always present in the current measurements. They can be clearly distinguished in DMT, adhesion, dissipation, energy dissipation, as well as peak-force maps. Areas with conductive spots exhibit high DMT (stiffness) values, which indicate a higher elasticity compared to the surrounding non-conductive areas. The ionic conductivity is accompanied by an upward water stream, which causes the high elasticity. Consequently, the stiffness should be high above the water-filled areas, as is visible in Figure 10e. Because the compressibility of liquid water is smaller than that of the polymer, the openings in the conductive area where water emerges are least deformable. The trend of small adhesion values at the area of the active conductive network can be explained with the help of the calibration measurements on the sample with different PTFE contents given in Figure 8. These measurements showed that a smaller adhesion signal (in dry/ambient environment) is associated with a lower PTFE content at the surface. We expect lower PTFE contents at the conductive areas where the sulfonic acid groups are present. In these conductive areas, the high stiffness values and the swelling are explained by a buildup of subsurface pressure due to the upward water stream. For geometric reasons, this subsurface pressure leads to a decrease in the surface fraction of hydrophobic back-bone in this area, as depicted in Figure 11. 

**Figure 12:**
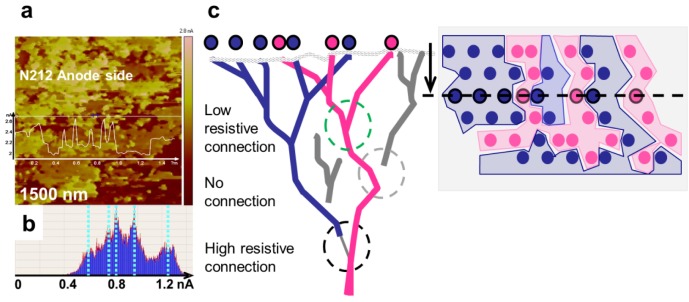
(**a**) PeakForce-QNM^®^ current of the Nafion^®^ 212 half-MEA at the anode side after 1600 h of operation at open-circuit voltage; (**b**) Histogram with distinct current level; (**c**) Schematic drawing of interpenetrating ionic subnets with a conductive, a highly resistive, or no subsurface connection.

The white spots inside these areas with high adhesion coincide with current spots. Small water droplets most likely decorate the openings of the conductive channel and lead to these high adhesion forces between water and the AFM tip, which has been demonstrated for a comparable material [23]. In most of the conductive network, no water droplets are visible at the surface, which can be explained by a faster evaporation of smaller droplets due to the considerable heating at current densities of 0.8 nA associated with heat generation from the reaction enthalpy of water formation. Another possible explanation originates from the finite sampling steps, where the maximal adhesion force might not be measured if the tip does not touch the channel centrally. The difference in the mechanical properties of conductive and non-conductive areas is caused by the existence of water pressure associated with the current flow. In all of the measurements, heterogeneity of the conductivity was observed and statistically distributed areas were always found without any current. No principle argument can be made for a chemical and structural difference on a scale much larger than the phase separation of Nafion^®^. Therefore, these observed non-conductive areas might be part of a non-activated subnet that may have a high resistive connection to a conductive part of the network; alternatively, it may also indicate an isolated subnet. In other measurements, an indication of the existence of differently conductive subnets was observed. In this case, a highly conductive membrane measured after fuel cell operation exhibited large conductive areas with a few different defined current levels, and the conductive areas formed an interpenetrating network [32]. In Figure 12a, the current image at the inner interface of the anode side of a N212 half-MEA measured after disassembly is shown [33]. As evident in the current histogram (Figure 12b), a few distinct current levels are observed that interpenetrate each other. A possible explanation is given in the scheme in Figure 12c. Distinct current levels indicate connected subnets, which have either no connection or a highly resistive connection beneath the surface. The existence of areas without any current even after operation of the fuel cell may indicate that, during operation, some parts of the membrane are not participating in the conduction. These parts may be a subnet that is completely isolated from a continuous network. Conceivably, under steady-state conditions, some areas may stay dry because a low-resistive network has been established that does not change at larger scale or because subnets exist that do not have a connection to a conductive path. We have previously observed that higher humidity and temperatures decrease the size and density of non-conductive areas [34,35]. At present, it has not been possible to verify if a certain part of the surface area is still non-conductive under fuel cell operating conditions of 60–80 °C. Measurements conducted at the operating temperature, which are underway, may clarify this matter.

### 3.4. Time Dependence of Conductive Channels

**Figure 13:**
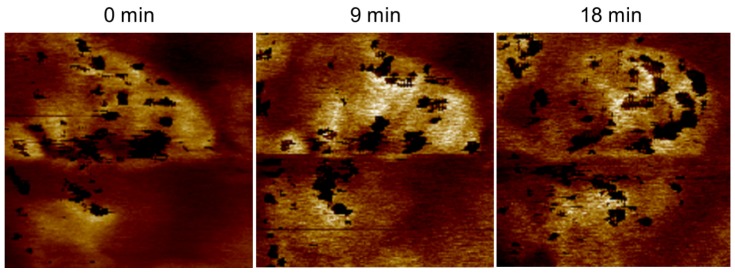
AFM stiffness and subtracted PeakForce-TUNA^®^ current image of the same area of activated Nafion^®^112 measured one after the other with an acquisition time of 9 min. The size of the swelling and the position and size of current spots change with time.

Figure 13 shows the stiffness (DMT modulus) maps with subtracted current images (black spots). The size of the conductive area (high stiffness) increases with time from a diameter of 115 to 140 nm after 9 min and to 190 nm after 18 min. Upon closer inspection, the black spots inside this area change with time. Obviously, the conductive openings at the surface are also changing with time. As evident in the stiffness image, the stiffest areas are located between the conductive spots. Directly at the position of current flow, the stiffness is somewhat smaller. Based on these observations, the existence of a conductive subnet connected beneath the surface can be concluded. The change in the conductive spots may then be interpreted as a frequent change in the actual conductive branch of this subnet: under high pressure of water from beneath (no current flow yet); a high stiffness first occurs before an opening is created at a certain pressure. Subsequently, under current/water flow, the pressure decreases, and, after a certain time, the opening may close; at this point, the current/water now emanates from a neighboring branch. The closing may be caused by a pressure drop due to another active branch or due to drain from the conductive network. The observation of a finite dynamic in the conductive network, even at room temperature, has been previously reported [23]. The size of the whole area increases with the pressure of the water flow. When the membrane dries due to diminished water supply from the back electrode, the swelling of the conductive area decreases almost completely; however, some of the conductive areas remain active until the conductivity drops completely.

### 3.5. Comparison of Nanostructure Observations with Literature Models

**Figure 14:**
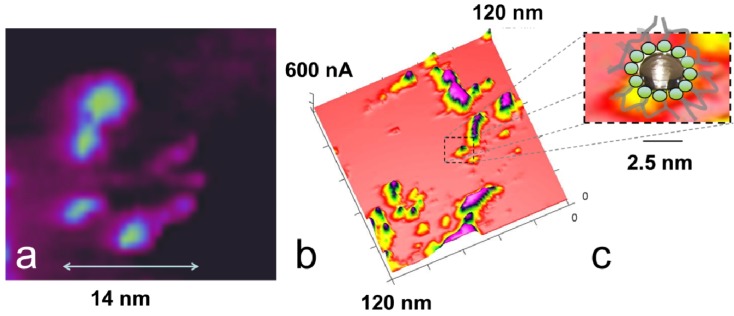
(**a**) AFM PeakForce-TUNA^®^ ionic current image of activated Nafion^®^ 112 with a spiral nanostructure with partly connected current spots. (**b**) Larger area of same ionic current measurement in (**a**) with linear current structures as well as single current spots. (**c**) A schematic drawing of an inverted micelle with a size of 2.5 nm according to the model of Schmidt-Rohr is superimposed onto a single current spot.

Two different primary models have been recently discussed in the literature. Based on small-angle X-ray scattering (SAXS) measurements, Schmidt-Rohr proposed the existence of parallel water channels formed from polymer backbones that provide a 1-D water channel inside the membrane. These inverted micelles with interior sulfonic acid groups have a diameter of approximately 4 nm with a mean diameter of 2.4 nm for the interior water channel. The other model, proposed by Gebel and Diat [11,12,13,14,15], is also based on SAXS and small-angle neutron scattering (SANS). They propose cylindrical fibrils of polymer backbone with 4 nm thick fibrils that are assembled in differently oriented bundles of approximately 50 nm and are embedded in water as a matrix. The sulfonic acid groups are situated outside the fibrils. MD model calculations of dry Nafion^®^ polymer chains show arrangement in such a manner after the introduction of water. The surface protrusions that form the step edges that are visible in, e.g., Figure 6 and Figure 9, may be identified as polymer backbone bundles with a size of 50 nm, as proposed by the model of Gebel and Diat [11,12,13,14,15]. The steps are then formed by aligned parallel polymer fibrillar bundles, which are assembled like sheets or lamellae and are orientated with an angle of approximately 13° ± 3° to the (flat) surface determined from 15 features. An example is depicted in the right image in Figure 7. The steps are obviously openings of the conductive channels where water associated with the sulfonic acid groups between them allow a protonic current flow. Because at the surface the current/water channel must be closed by the polymer backbone, a distinction between inside or outside the sulfonic acid groups is not possible. The inside structure of these sheets cannot be resolved. According to Schmidt-Rohr [10], isolated protonic current spots may indicate the existence of isolated parallel conductive channels. Some isolated current spots, current spots aligned like curved chains, as well as elongated conductive areas are visible in Figure 14b. 

Figure 14a shows a spiral conductive nanostructure with a diameter of 14 nm formed by partly connected and partly isolated ionic current spots with a diameter that decreases from 5.2 nm for the larger left four spots and to approximately 1.6 nm for the spots on the right side. The two bright current spots are separated by 1.5 nm, and a small spot is located between them. The diameter of the non-conductive center is approximately 4–5 nm. This structure could be interpreted as being formed from a cylindrical fiber of polymer backbone with exterior sulfonic acid groups, most likely surrounded by other fibrillar bundles of polymer backbone. The space is filled with water and displays current spots. These spots can be differentiated by some highly active current spots but appear to be connected by a smaller current. The different orientation and density of the sulfonic acid groups outside the fibrils may lead to the formation of more or fewer connected conductive water channels between them. Although their diameter fits the model prediction of Schmidt-Rohr [10], their conductive connection is in disagreement with this model. These spirally shaped structures are frequently found in our current images of Nafion^®^. Linear structures are also visible in Figure 14b with no isolated current spots but an approximately homogeneous current. This observation indicates the presence of water sheets, which also fits the model proposed by Gebel and Diat [12]. The existence of elongated water sheets is also supported from the time-dependent measurements in Figure 13: The conductive spots visible after 18 min are partly connected and form curved lines, which is consistent with the inclusion of water between sheets of the polymer backbone.

According to the model of Schmidt-Rohr [10], single inverted micelles that can cluster together into hexagonal structures would be expected where the different water channels are completely included into a polymer backbone cylinder. The existence of single current spots, which could be interpreted as a single inverted micelle, may support this model. In Figure 14c, an isolated current spot with a size of 2.5 nm is compared to a scheme of this model and fits well. Based on the present measurements, the model proposed by Gebel and Diat is consistent with almost all ionic current density distributions and mechanical properties observed on Nafion^®^, whereas the model with cylindrical inverted micelles is unable to explain the majority of our observations. 

## 4. Conclusions

In this paper, the mechanical properties, especially the adhesion force and the DMT modulus (stiffness), were compared to the conductivity images at the nano-scale for a Nafion^®^ 112 solid-electrolyte membrane. One important factor concerning the nano-scale properties is the electro-osmotic drag, which always leads to a water flow associated with the protonic current. 

The activation of membranes by a forced current flow during a voltage step applied in the AFM setup was monitored over 35 s and indicated a characteristic step current increase with decaying transients. The time dependence of the current was interpreted as stepwise widening of a visco-elastic polymer channel with a stepwise current increase. Under an applied voltage, larger conductive channels are formed by such a process, and these channels may then fulfill the size requirement for hydrodynamic water flow.

After activation of the membranes, the existence of swollen areas (100–300 nm) with high stiffness and low adhesion and deformation values was observed, and conductive spots (1–10 nm) were restricted to theses parts of the surface. These areas were interpreted as being ionically conductive subnets connected beneath the surface. The water flow connected to the ionic current leads to a buildup of water pressure and subsequent swelling. The results are bumps with high stiffness, and the stiffness changes with time and with the size of the bumps. A frequent change in the position of active current spots was observed and was also explained by a change in the water pressure and changes in resistivity in different branches of the active conductive subnet. The decreased adhesion at swollen areas was explained by a decrease in the fraction of Nafion^®^ polymer backbones at the surface caused by expansion of the polymer area above the conductive subnet. 

With the assumption that the existence of active conductive subnets and subnets are not active because of, e.g., a highly resistive connection, the generally observed heterogeneity of conductivity can be explained. The observation of a change in the water/current flow under an applied voltage leads to the assumption that, at the time of voltage application and current flow, the water flow and the subsequent pressure form an active conducting network according to a path of lowest resistance, which should exhibit a preferential orientation perpendicular to the surface.

The observed nanostructures are in agreement with a model in which bundles of fibrils with a diameter of approximately 50 nm from the molecular backbone are present. Ionic current is detected as spots that are more or less connected to form chains or sheets; these spots indicate the existence of water sheets or films. In conclusion, the existence of single inverted micelles, as proposed by Schmidt-Rohr [10], is possible and cannot be excluded; however, a significantly better agreement was observed between the observed results and the model of Diat and Gebel [11,12,13,14,15].

## References

[1] Alberti G., Narducci R., Sganappa M. (2008). Effects of hydrothermal/thermal treatments on the water-uptake of Nafion membranes and relations with changes of conformation, counter-elastic force and tensile modulus of the matrix. J. Power Sources.

[2] Eisenberg A. (1970). Clustering of ions in organic polymers. A theoretical approach. Macromolecules.

[3] Zhao Q., Majsztrik P., Benziger J. (2011). Diffusion and interfacial transport of water in Nafion^®^. J. Phys. Chem. B.

[4] Satterfield M.B., Benziger J.B. (2008). Non-Fickian water vapor sorption dynamics by Nafion membranes. J. Phys. Chem. B.

[5] Bass M., Berman A., Singh A., Konovalov O., Freger V. (2010). Surface structure of Nafion^®^ in vapor and liquid. J. Phys. Chem. B.

[6] Bass M., Berman A., Singh A., Konovalov O., Freger V. (2011). Surface-induced micelle orientation in Nafion films. Macromolecules.

[7] Gierke T.D., Munn G.E., Wilson F.C. (1981). The morphology in nafion perfluorinated membrane products, as determined by wide- and small-angle X-ray studies. J. Polym. Sci. Polym. Phys..

[8] Hsu W.Y., Gierke T.D. (1982). Elastic theory for ionic clustering in perfluorinated ionomers. Macromolecules.

[9] Hsu W.Y., Gierke T.D. (1983). Ion transport and clustering in nation perfluorinated membranes. J. Membr. Sci..

[10] Schmidt-Rohr K., Chen Q. (2008). Parallel cylindrical water nanochannels in Nafion fuel-cell membranes. Nature Mater..

[11] Gebel G. (2000). Structural evolution of water swollen perfluorosulfonated ionomers from dry membrane to solution. Polymer.

[12] Rubatat L., Rollet A.L., Gebel G., Diat O. (2002). Evidence of elongated polymeric aggregates in Nafion. Macromolecules.

[13] Rollet A.L., Gebel G., Diat O. (2002). A new insight into Nafion structure. J. Phys. Chem. B.

[14] Rubatat L., Gebel G., Diat O. (2004). Orientation of Drawn Nafion at Molecular and Mesoscopic Scales. Macromolecules.

[15] Rubatat L., Gebel G., Diat O. (2007). Stretching effect on Nafion fibrillar nanostructure. Macromolecules.

[16] Mauritz K.A., Moore R.B. (2004). State of Understanding of Nafion. Chem. Rev..

[17] Rao V., Friedrich K.A., Stimming U., Pabby A.K., Rizvi S.S.H., Sastre A.M. (2008). Part III: Membrane Applications in Industrial Waste Management (including nuclear Nuclear), Environmenatal Engineering and Future Trends in membrane Science Applications in Industrial Waste Management (Including Nuclear), Environmental Engineering and Future Trends in Membrane Science. Handbook of Membrane Separations: Chemical, Pharmaceutical, Food, and Biotechnological Applications.

[18] Smitha B., Sridhar S., Khan A.A. (2005). Solid polymer electrolyte membranes for fuel cell applications-A review. J. Membr.Sci..

[19] Anantaraman A.V., Gardner C.L. (1996). Studies on ion-exchange membranes. Part 1. Effect of humidity on the conductivity of Nafion. J. Electroanal. Chem..

[20] Vol’fkovich Yu.M., Sosenkin V.E., Nikol’skaya N.F. (2010). Hydrophilic-hydrophobic and sorption properties of the catalyst layers of electrodes in a proton exchange membrane fuel cell: A stage by stage study. Russ. J. Electrochem..

[21] Vol’fkovich Yu.M., Sosenkinz V.E., Nikol’skaya N.F. (2010). Hydrophilic-hydrophobic and sorption properties of the catalyst layers of electrodes in a proton-exchange membrane fuel cell: A stage-by-stage study. Elektrokhimiya.

[22] Derjaguin B.V., Muller V.M., Toporov Y.P. (1975). Effect of contact deformations on the adhesion of particles. J. Colloid Interface Sci..

[23] Hiesgen R., Wehl I., Aleksandrova E., Roduner E., Bauder A., Friedrich K.A. (2010). Nanoscale properties of polymer fuel cell materials-A selected review. Int. J. Energy Res..

[24] Hink S., Wagner N., Bessler W., Roduner E. (2012). Impedance spectroscopic investigation of proton conductivity in Nafion using transient electrochemical atomic force microscopy (AFM). Membranes.

[25] Roduner E., Hiesgen R., Garche J., Dyer C., Moseley P., Ogumi Z., Rand D., Scrosatti B. (2009). Spatially resolved Measurements. Encyclopedia of Electrochemical Power Sources.

[26] Hiesgen R., Haiber J., Garche J., Dyer C., Moseley P., Ogumi Z., Rand D., Scrosatti B. (2009). Structural properties: Atomic force microscopy. Encyclopedia of Electrochemical Power Sources.

[27] O'Dea J.R., Buratto S.K. (2011). Phase Imaging of Proton Exchange Membranes under Attractive and Repulsive Tip Sample Interaction Forces. J. Phys. Chem. B.

[28] Sanchez D.G., Diaz D.G., Hiesgen R., Wehl I., Friedrich K.A. (2010). Oscillations of PEM fuel cells at low cathode humidification. J. Electroanal. Chem..

[29] Gebel G., Lyonnard S., Mendil-Jakani H., Morin A. (2011). The kinetics of water sorption kinetics in Nafion^®^ membranes: A small-angle neutron scattering study. J. Phys. Conds. Matter.

[30] Choi P., Jalani N.H., Datta R. (2005). Thermodynamics and proton transport in Nafion^®^. II. Proton diffusion mechanisms and conductivity. J. Electrochem. Soc..

[31] Haubold H.G., Vad T., Jungbluth H., Hiller P. (2001). Nano structure of Nafion: A SAXS study. Electrochim. Acta.

[32] Zhang S., Yuan X.Z., Hiesgen R., Friedrich K.A., Wang H. (2012). Effect of open circuit voltage on degradation of a short proton exchange membrane fuel cell stack with bilayer membrane configurations. J. Power Sources.

[33] Yuan X.Z., Zhang S., Ban S., Huang C., Wang H., Singara V., Fowler M., Hiesgen R., Schulze M., Haug A., Friedrich K.A. (2012). Degradation of a PEM fuel cell stack with Nafion^®^ membranes of different thicknesses. Part II: *Ex situ* diagnosis. J. Power Sources.

[34] Aleksandrova E., Hiesgen R., Eberhard D., Friedrich K.A., Kaz T., Roduner E. (2007). Nanometer scale visualization of ionic channels at the surface of a proton exchange membrane. ChemPhysChem.

[35] Aleksandrova E., Hiesgen R., Eberhard D., Friedrich K.A., Roduner E. (2007). Electrochemical atomic force microscopy study of proton conductivity in a Nafion^®^ Membrane. Phys. Chem. Chem. Phys..

